# Case Report: Reconstruction After Anterior Pubic Hemipelvectomy

**DOI:** 10.3389/fsurg.2021.585600

**Published:** 2021-05-21

**Authors:** Benjamin Murphy, Tharsa Thillainadesan, Kerian Robinson, Anita Clarke, Peter Choong

**Affiliations:** ^1^Department of Orthopaedic Surgery, St Vincent's Hospital, Melbourne, VIC, Australia; ^2^Department of Urology, St Vincent's Hospital, Melbourne, VIC, Australia; ^3^Department of Surgery, The University of Melbourne, Melbourne, VIC, Australia

**Keywords:** reconstruction of anterior pelvis, anterior hemipelvectomy, type III hemipelvectomy, pubic resection, chondrosarcoma

## Abstract

We report on a case of a large atypical cartilaginous tumor of the pelvis and its novel surgical resection with an anterior hemipelvectomy and reconstruction with an iliac crest graft. Surgical intervention is the mainstay treatment of pelvic chondrosarcomas. However, there have been reports of concern regarding preventing pelvic visceral herniation and adequately reconstructing the pelvis. This report is unique within the literature and has yielded good functional outcomes whilst achieving satisfactory surgical margins and minimizing morbidity.

## Introduction

Chondrosarcomas are malignant tumor of mesenchymal origin whose cells produce osteoid-free chondroid matrix and can arise *de novo* or secondary to a pre-existing benign cartilaginous neoplasm (1–4). They are the second most common primary bone tumor, after osteosarcoma, and are most commonly found within the pelvis and proximal femur (1, 5, 6). Chondrosarcomas exist along a continuum of severity with low grade lesions exhibiting an indolent growth pattern, whilst high grade lesions readily metastasize and have a poor prognosis (2, 6). Patients almost always present with insidious and progressive pain, usually for months to years and especially at night (1, 2). Other common presentations include a palpable lump, pathological fracture and mass-effect symptoms including nerve impingement (1, 2).

Surgical resection has been the mainstay of treatment for these tumors as their abundant extracellular matrix, poor vascularity and low percentage of actively dividing cells render the tumors chemotherapy and radiotherapy resistant (4, 7). The surgical management of pelvic chondrosarcomas needs to involve en-bloc resection, especially for high grade lesions, as intralesional resection has been demonstrated to be ineffective (8–10). Therefore, operating surgeons must balance the risk of significant morbidity against the outcomes of definitive resection with options including hip disarticulation vs. more conservative limb sparing operations including internal hemipelvectomy (2). Internal hemipelvectomy can be classified according to resection site into type 1 (ilium), type 2 (periacetabular) and type 3 (pubis) with this case involving a type 3 resection of both left and right pubic bones (11).

Herein, we report on a novel technique in the surgical management of a large pelvic atypical cartilaginous tumor via anterior hemipelvectomy with iliac crest graft reconstruction. The tumor was classified as an atypical cartilaginous tumor emerging from the posterior aspect of the pubis causing significant displacement and obstruction of the pelvic viscera without any local infiltration. Due to the large mass resected, there was a need for reconstruction to prevent herniation which was achieved via computer-navigated resection followed by computer guided iliac crest autograft harvest. This case highlights key principles in orthopedic oncology as well as providing a unique approach to the management of a complex case.

## Case Description

A 54-year-old nulligravida female had a 10-year history of nocturia, urinary frequency and recurrent urinary tract infections which had been managed with short courses of antibiotics. On presentation, physical examination revealed a firm hard mass along the anterior vaginal wall. Her examination was otherwise unremarkable and specifically no evidence of bony dysplasia of her limbs. Despite the length of symptoms, she had not undergone any investigations for her long-standing urinary symptoms until a computed tomography (CT) scan of her pelvis was organized and a large pelvic mass emerging from her pubis was noted, prompting specialist sarcoma service referral. The patient consented to the production of this report and it was approved by our ethics committee.

### Timeline

See Figure 1.

**Figure 1:**
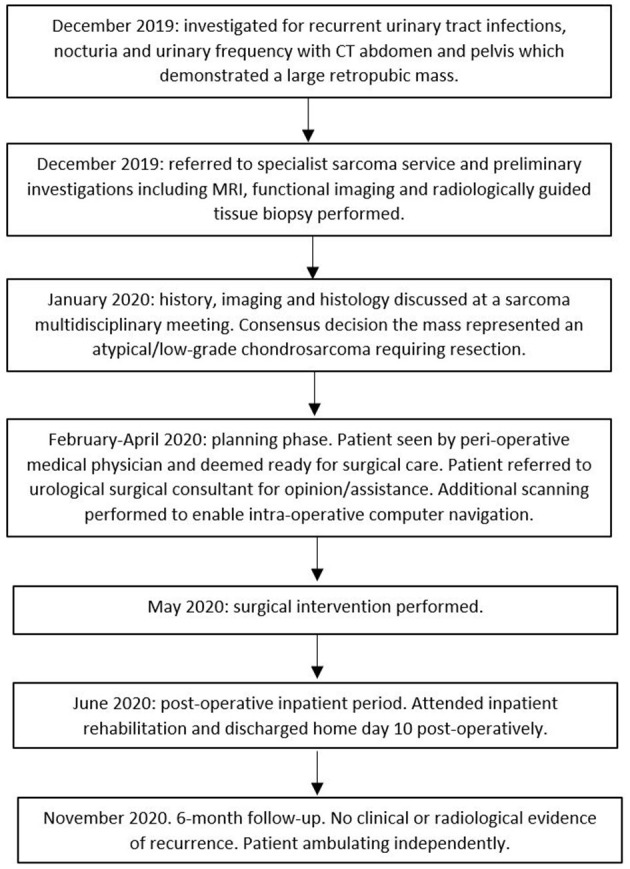
Timeline of case report.

#### Diagnostic Assessment

In addition to an abdominal and pelvic CT scan, the patient underwent further staging studies. These included a magnetic resonance imaging (MRI) which demonstrated a large heterogenous T2 bright lesion measuring 9 ×6 cm emerging from the posterior aspect of the right body of the pubis. The mass projected postero-superiorly with a large, 1 cm thick cartliagenous cap and an underlying stalk that communicated with the medullary cavity of the pelvis. There was also significant effacement of the bladder with compression of the right ureter and resulting hydronephrosis. There was no infiltration of any surrounding structures.

Functional imaging including both thallium-201 and technetium-99 pentavalaent dimercaptsouccinic acid (DMSA V) scintigraphy were utilized and demonstrated minimal metabolic activity within the tumor. A CT scan of the chest was performed which excluded pulmonary metastatic disease. Following these investigations, a CT-guided tissue biopsy targeting an area of increased metabolic activity demonstrated a cartilaginous lesion with mild atypia and some focal calcification. This case was discussed at a multidisciplinary meeting involving sarcoma specialist surgeons, radiologists and pathologists. Given the size and histological nature of the lesion, the decision was made to manage this lesion as an atypical cartilaginous tumor.

#### Therapeutic Intervention—Surgery

**Figure 2:**
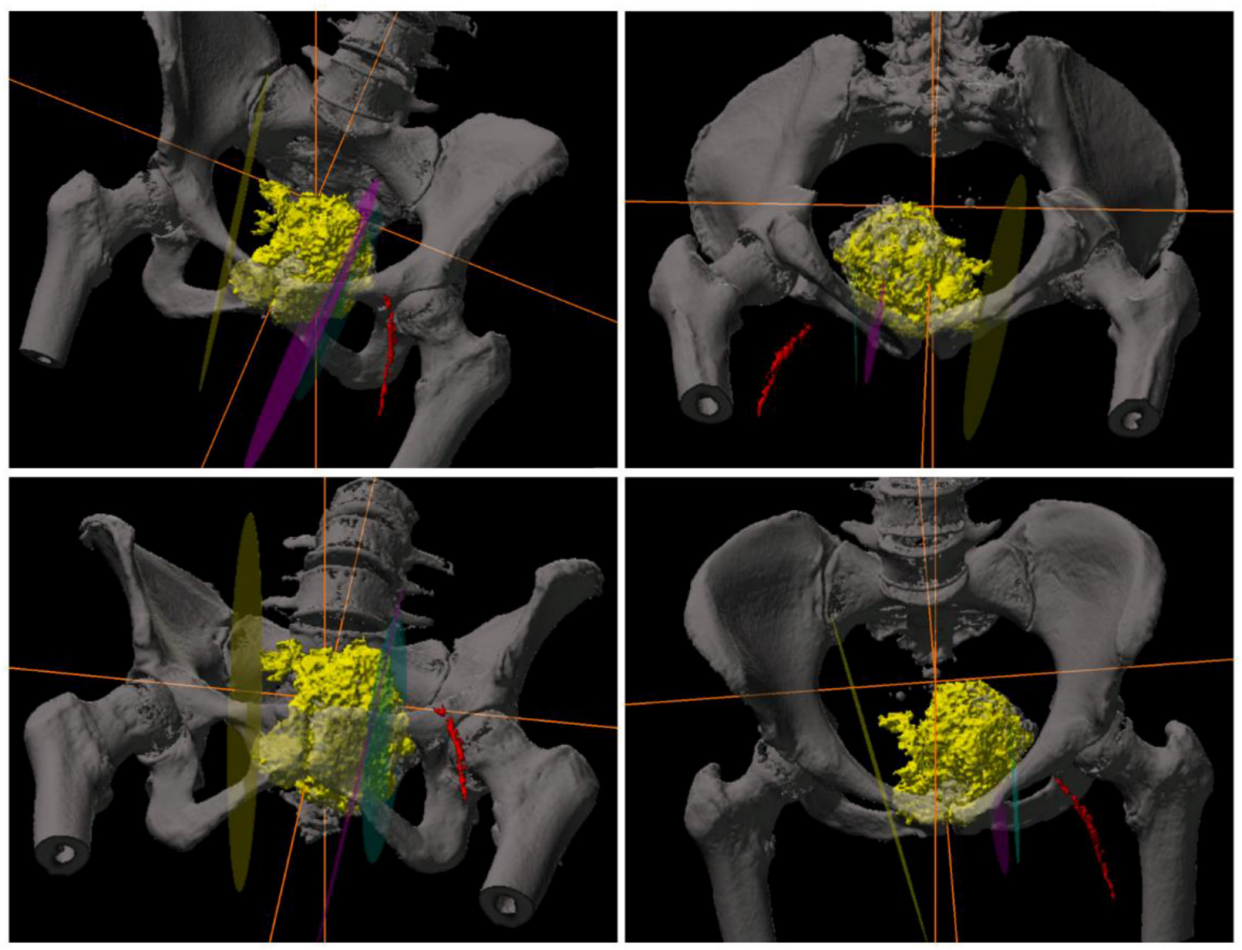
3D-CT reconstruction images of the pelvis demonstrating the tumor and computer navigation-system utilized intra-operatively.

**Figure 3:**
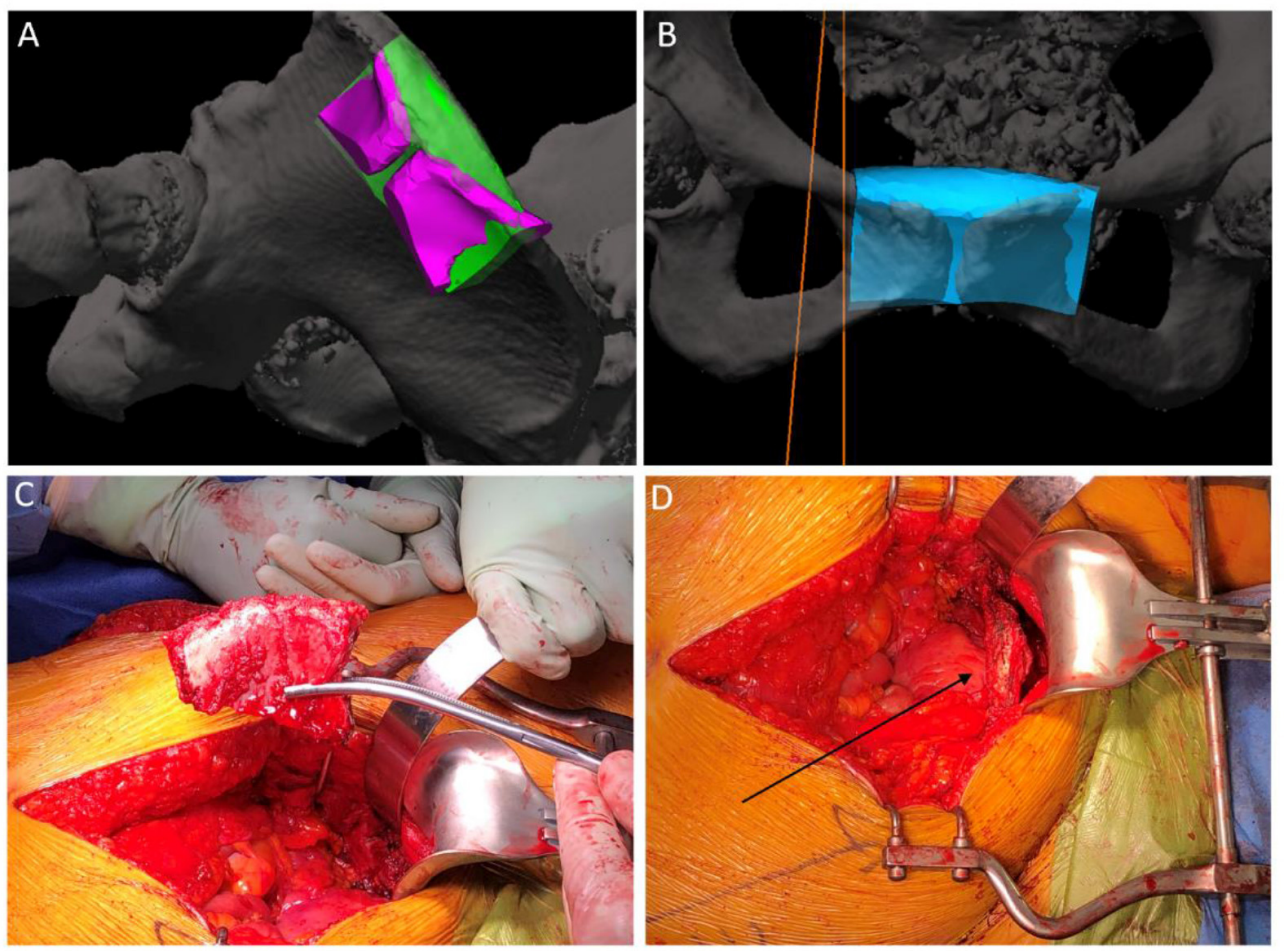
Intra-operative photos. **(A)** demonstration of the computer-navigated iliac crest resection with pubic cut overlay. **(B)** demonstration of iliac crest craft positioning. **(C)** iliac crest graft post resection. **(D)** Arrow demonstrating positioning of iliac crest graft in the resected pubis.

This surgical intervention represents a unique approach to the management of a complex chondrosarcoma of the pelvis. The patient was given a general anesthetic and positioned in supine. The procedure commenced with a cystoscopy, bilateral retrograde pyelography and insertion of a left ureteral stent to alleviate obstruction. Significant distortion of the bladder and urethra were noted secondary to the external compression from the tumor. There was no local infiltration of the tumor into surrounding structures. The tumor was then approached via a longitudinal lower midline abdominal incision. Meticulous haemostasis was undertaken whilst dissection was performed to the level of the pubic body. This allowed the displacement of the pelvic viscera from around the tumor within the lesser pelvis, and detachment of the origins of the adductor musculature from the anterior external pelvic wall. At this stage, navigation pins were inserted into the iliac crest and registration of important bony pelvic landmarks with the computer navigation system was confirmed (Stryker Navigation System with Spinemap 3D 3.1 software®) (12) (Figure 2). Using navigation, the planned osteotomies were created through bilateral superior and inferior pubic rami to mobilize the tumor (Figures 3A,B). The tumor was carefully dissected from the bladder neck by rolling it forward away from the female pelvic viscera and surrounding internal pelvic neurovasculature and ultimately resected en-bloc from the patient without complication. Using the same navigation system, a preplanned segment of iliac bone was marked out on the ilium. This segment was matched in size and curve to the resected pubic bone using a pre-operative computer model. Osteotomy of the iliac graft was then computer-navigated (Figure 3C). The iliac crest graft was prepared with drill holes and secured with Arthrex fiber wire sutures (Arthrex FiberTape 2 mm ®) to the adjacent pubic rami (Figure 3D) (13). The right and left rectus abdominis musculature were sutured to the released ends of the right and left gracilis muscles. Two surgical suction drains were placed, one at the pubic resection site and the other at the iliac bone resection site. During the operation, the patient was transfused 4 units of packed red blood cells and 1 unit of fresh frozen plasma.

### Follow-Up and Outcomes

**Figure 4:**
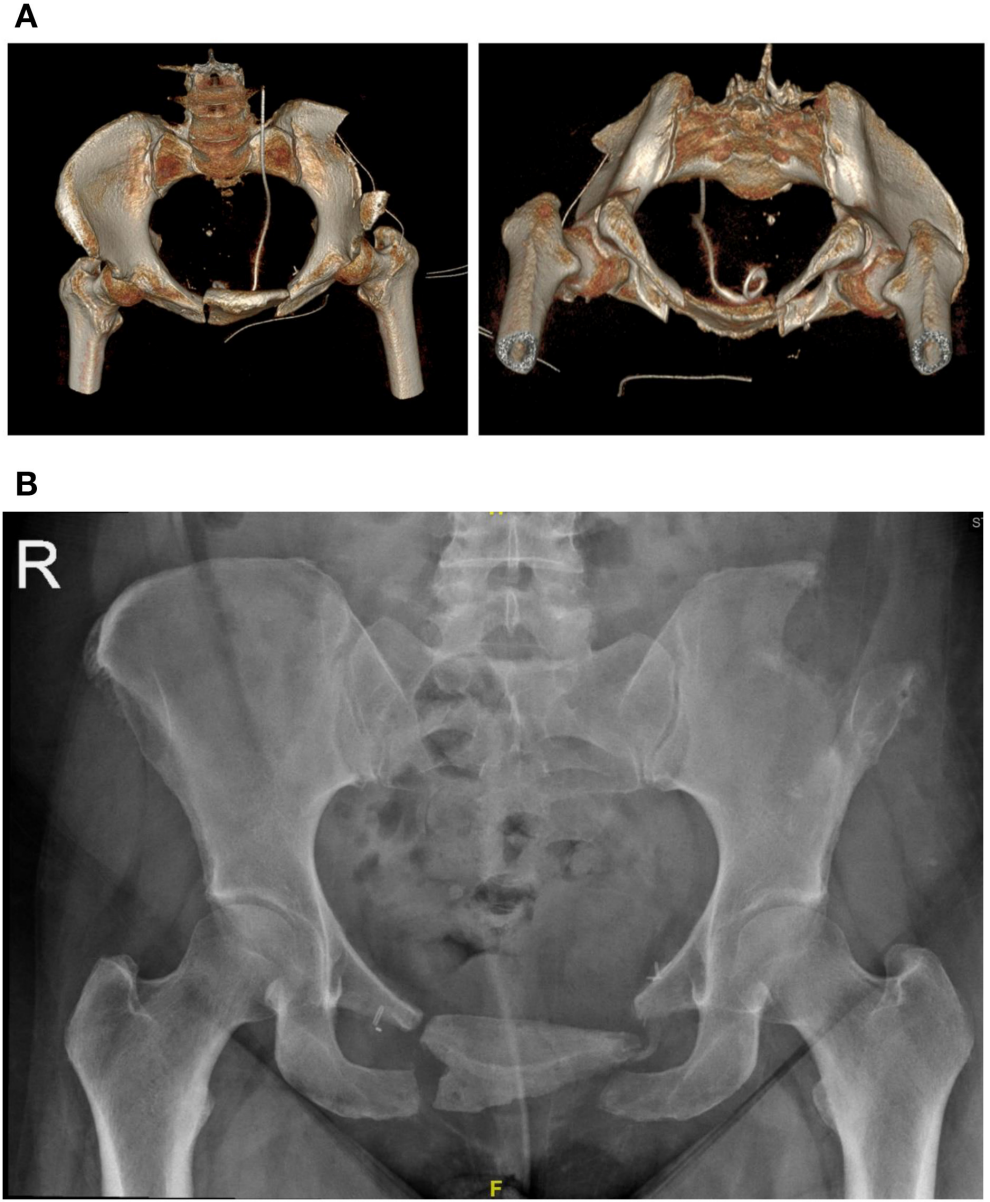
**(A)** day 1 post surgery. 3D reconstructions of a CT pelvis demonstrating the iliac crest graft *in-situ*. **(B)** 6-month follow up X-Ray demonstrating stable positioning of the graft.

Post-operatively, the patient was allowed to fully weight bear and had a post-operative course consistent with a grade II Clavien-Dindo surgical complication (i.e., minor alteration to management) (14). She was successfully treated for a post-operative urinary tract infection, presumably related to her Foley catheter, with culture-specific oral antibiotics. Post-operative scans demonstrated adequate positioning of the graft with no immediate complications (Figure 4). The resected retropubic mass was confirmed to be a grade one chondrosarcoma/atypical cartilaginous tumor on histopathological analysis. There was a >1 mm clear margin circumferentially around the tumor which was completely intact with a thick fibrous capsule. She was discharged from the acute surgical ward to a local rehabilitation hospital on day 7. At the time of discharge home from the rehabilitation ward, the patient was able to ambulate independently with a frame for 30 meters and rise from supine to standing independently At the 2-week post-operative mark her surgical wounds were healing well with no signs of infection. At the 6-week post-operative mark, the patient was ambulating well with two crutches and had normal bowel and urinary continence. She still described some pain and discomfort at her pubis with a sense of decreasing movement and discomfort of the grafted area. At 6-month follow-up there was no clinical or radiological evidence of tumor recurrence and she was able to ambulate independently (Figure 4).

## Discussion

This case report provides an insight into the diagnosis and management of pelvic chondrosarcoma as well as a novel approach in its surgical resection and reconstruction. It has been well-established within the literature that pelvic chondrosarcoma is difficult to diagnose pre-operatively (15). Firstly, whilst there are characteristic radiological appearances, it is difficult to distinguish chondrosarcomas from benign chondroid lesions (16–18). Crim et al. demonstrated only a 21 and 58% sensitivity of plain radiographs and MRI for accurately diagnosing grade 1 chondrosarcomas (19). Additionally histological diagnosis, especially in regards to pelvic chondrosarcoma, can be error-prone due to sampling misrepresentation and lack of consensus amongst experts (2, 15, 20). Tsuda et al. estimated this error margin to be as high as 63% (21). In our case, the initial histology and radiological investigations tended to favor a more benign cause such as osteochondroma. However, given the size and location of the tumor there was still a high index of suspicion that this tumor represented an atypical cartilaginous tumor and thus the treatment was tailored to this diagnosis.

Pelvic chondrosarcomas also represent a surgical challenge, with local recurrences rates being higher and the tumors conveying a poorer prognosis than peripheral bone chondrosarcomas (4, 5, 19, 20). There are few anatomical barriers to extension within the pelvis and therefore the tumors are often exceptionally large and with higher grade behavior (5, 6, 19). With our resection we achieved a wide surgical margin (i.e., >1 mm in all directions) for this grade 1 chrondrosarcoma/atypical cartilaginous tumor, whilst minimizing morbidity. In a multicenter study by Tsuda et al. this margin was considered the desired outcome for all pelvic chondrosarcomas as they can often be prone to inaccuracies in diagnosis and high local recurrence rates (21).

Type 3 hemipelvectomies are relatively uncommon and account for ~10% of internal hemipelvectomies (22, 23). Traditionally, it was considered unnecessary to reconstruct following type III hemipelvectomies given the preserved continuity of the weight bearing axis and the potential complications associated with reconstruction (24). However, Imanshi et al. in 2015 reported two case reports of type 3 internal hemipelvectomies in which bladder herniation was noted to be a significant concern following pubic resection and recommended reconstruction with a non-bony material (25). Similarly, von Rundstedt et al. and Arkoulis et al. both reported on cases of bladder herniation following type 3 internal hemipelvectomies (26, 27). The concern rising from these case reports is that previous reconstruction methods which relied on soft tissue have not provided enough rigidity or durability to prevent herniation. Whilst bone graft with internal fixation could be considered an option, the natural human pubic symphysis permits motion up to 2 mm of shift and 1 degree of rotation which would promote metal stress and potentially implant failure (28, 29). Therefore, we opted to use the iliac crest as bone graft and fix it via thick fiber wire sutures to allow sufficient post-operative movement to achieve a fibrous union much in the same way that the symphysis pubis is a fibrous joint. Computer navigation and preoperative 3D planning facilitated not only the planning and execution of the pubic osteotomies but also the accurate and safe harvest of iliac bone to reconstruct the defect.

## Conclusion

We report on a case of a large atypical cartilaginous tumor of the pelvis and its novel surgical resection with an anterior pubic hemipelvectomy and reconstruction with an iliac crest graft. This report is unique within the literature and has yielded good early functional outcomes whilst safely treating the malignancy.

### Patient Experience

Over many years I sought treatment from several different general practitioners for recurring urinary infections and urinary incontinence. It seemed to me that many courses of antibiotics were having only short-term effects. My urinary frequency had reached the point where urgent hourly visits to the toilet made work and sleep difficult.

Since my surgery I have observed reduced urinary frequency to only 2–3 times nightly and therefore much improved sleep. Also, I have much improved ease of bowel movements. My pain post-surgery was minimal and the use of strong pain medication only lasted 8 days. Additionally, after only 4 weeks, even with the extent of the surgery, I was able to move around the house unaided and walk for about 600 m in 20 min with the aid of my wheelie walker. Now, after 8 weeks I am walking over 1 km in 30 min also with the aid of my walker with current physio advice to start walking this distance unaided which I see as quite achievable.

## Data Availability Statement

The original contributions presented in the study are included in the article/supplementary material, further inquiries can be directed to the corresponding author/s.

## Ethics Statement

The studies involving human participants were reviewed and approved by St Vincent's Hospital (Melbourne) Human Research Ethics Committee (HREC). The patients/participants provided their written informed consent to participate in this study. Written informed consent was obtained from the individual(s) for the publication of any potentially identifiable images or data included in this article.

## Author Contributions

All authors listed have made a substantial, direct and intellectual contribution to the work, and approved it for publication.

## Conflict of Interest

The authors declare that the research was conducted in the absence of any commercial or financial relationships that could be construed as a potential conflict of interest.
